# The Moss Flora of Akdağ Mountain (Amasya, Turkey)

**DOI:** 10.1155/2014/860379

**Published:** 2014-12-23

**Authors:** Kerem Canli, Barbaros Çetin

**Affiliations:** Department of Biology, Faculty of Science, Dokuz Eylül University, 35390 Izmir, Turkey

## Abstract

The moss flora of Akdağ Mountain (Amasya, Turkey) was investigated. At the result of identifications of 1500 moss specimens, collected from the research area, 178 taxa belonging to 69 genera and 26 families were determined. Among them, 94 taxa are new for A3 grid square according to the Turkey grid system which was adopted by Henderson. The location data of *Grimmia crinitoleucophaea* Cardot and *Barbula enderesii* Garov. are the first records for Turkey, and *Encalypta spathulata* Müll. Hal., *Schistidium dupretii* (Thér.) W. A. Weber, *Weissia condensa* var. *armata* (Thér. & Trab.) M. J. Cano, Ros & J. Guerra, *Tortella bambergeri* (Schimp.), *Barbula enderesii* Garov., *Hedwigia ciliata* var. *leucophaea* Bruch & Schimp., and *Campyliadelphus elodes* (Lindb.) Kanda are recorded for the second time to the byroflora of Turkey.

## 1. Introduction

Turkey, which is in the transition zone of three biogeographical regions, the Mediterranean, European-Siberian, and Irano-Turanian, is one of the richest countries between the Middle East and Europe in terms of biodiversity [1]. Unfortunately, knowledge of the Turkish bryoflora is still far from complete. To date, neither Turkish nor foreign bryologists have visited some regions, especially south-eastern Turkey. However, some recent additions with increasing research activities indicate that quite a number of new discoveries may be expected. In recent years, the studies on moss biodiversity of Turkey have increased and are enriched with many new findings. Some additions to the moss flora of Turkey in the last years include* Didymodon tomaculosus* (Blockeel) M. F. V. Corley [2],* Schistidium sordidum* Hagen. [3],* Bryoerythrophyllum rubrum* (Jur. ex Geh.) P. C. Chen [4],* Seligeria donniana* (Sm.) Müll. Hal. [5],* Conardia compacta* (Drumm. ex Mull. Hal.) H. Rob. and* Didymodon icmadophilus* (Schimp. ex Müll. Hal.) K. Saito [6],* Seligeria trifaria* (Brid.) Lindb. and* Pseudotaxiphyllum elegans* (Brid.) Z. Iwats. [7],* Dicranella schreberiana* (Hedw.) Dixon,* Dicranodontium asperulum* (Mitt.) Broth., and* Campylopus pyriformis* (Schultz) Brid. [8],* Grimmia anomala* Schimp.,* Pohlia filum* (Schimp.) Mårtensson, and* Hookeria acutifolia* Hook. & Grev. [9],* Sphagnum contortum* K. F. Schultz,* Sphagnum fallax* (H. Klinggr.) H. Klinggr.,* Sphagnum magellanicum* Brid.,* Sphagnum rubellum* Wilson [10], and* Sphagnum molle* Sull. [11].

**Figure 1:**
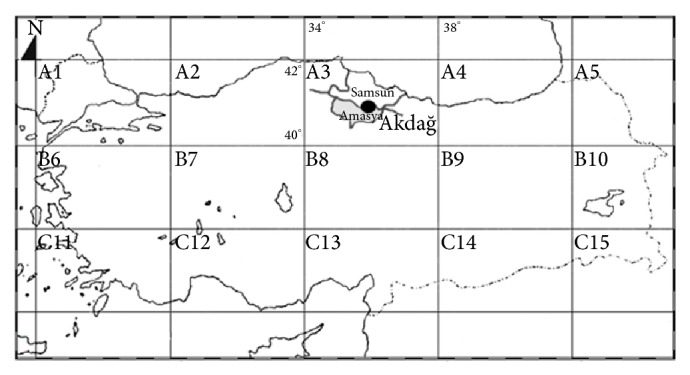
Amasya Akdağ and Henderson's grid system.

**Figure 2:**
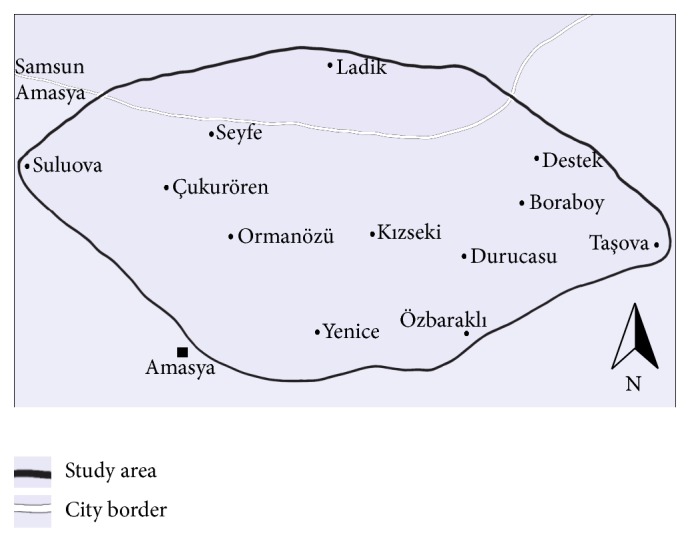
Study area.

Akdağ (Amasya), chosen as the study area, is located between Central Anatolia and the Black Sea region within the A3 square according to Henderson's [12] grid system (Figures 1 and 2). Although Akdağ Mountain has been named as one of the most important flora regions of Turkey, the moss flora of the mountain has not been studied before.

## 2. Materials and Methods

Samples were collected from 37 stations containing different habitats (Table 1), between 2009 and 2011. All specimens were deposited in the Herbarium of Ankara University (ANK), Faculty of Science, Department of Biology, Ankara.

The specimens were identified using relevant literature [13–37].

## 3. Results and Discussion

The new records for the A3 grid square are indicated with a single asterisk, the taxa recorded from Turkey for the second time with double asterisks, and the first location data from Turkey with triple asterisks in the floristic list. Station numbers are indicated in parentheses. The bryofloristic list was arranged according to Hill et al. [38] and revised with the latest accepted names according to Ros et al. [39]:

 (∗)** Timmiales (M. Fleisch.) Ochyra**
 (∗)** Timmiaceae Schimp.**
 (∗)** 1.* Timmia *Hedw. **
 (∗)* 1. Timmia bavarica *Hessl. (20, 21)
 
**Encalyptales Dixon**
 
**Encalyptaceae Schimp. **
 
**2.* Encalypta* Hedw.**
 
*2. Encalypta streptocarpa* Hedw.  (2, 16, 20, 21, 26, 33)
 (∗)* 3. E. intermedia* Jur.  (18)
 (∗)* 4. E. rhaptocarpa* Schwägr.  (15)
 (∗) (∗∗) 5.* E. spathulata* Müll. Hal.  (34)
 
*6. E. vulgaris* Hedw.  (26, 34)
 
**Funariales M. Fleisch.**
 
**Funariaceae Schwägr. **
 3. ***Entosthodon* Schwägr.**
 (∗) 7.* Entosthodon muhlenbergii* (Turner) Fife  (2)
 
**4. Funaria Hedw.**
 
*8. Funaria hygrometrica* Hedw.  (1, 34)
 
**Grimmiales M. Fleisch. **
 
**Grimmiaceae Arn. **
 
**5.* Grimmia* Hedw.**
 (∗) (∗∗∗)* 9. Grimmia crinitoleucophaea* Cardot  (10, 13, 25, 34)
 (∗)* 10. G. funalis* (Schwägr.) Bruch & Schimp.  (13)
 (∗)* 11. G. laevigata* (Brid.) Brid.  (7, 8, 13, 31, 34)
 (∗)* 12. G. montana* Bruch & Schimp.  (25)
 (∗)* 13. G. orbicularis* Bruch ex Wilson  (3, 7, 10, 23, 33)
 
*14. G. ovalis* (Hedw.) Lindb.  (25)
 
*15. G. pulvinata* (Hedw.) Sm.  (1, 2, 3, 10, 13, 15, 25, 32, 34, 35, 36)
 
*16. G. tergestina* Tomm. ex Bruch & Schimp.  (8, 25, 31)
 
*17. G. trichophylla* Grev.  (3, 5, 8, 36)
 
**6.* Schistidium* Bruch & Schimp.**
 
*18. Schistidium apocarpum* (Hedw.) Bruch & Schimp.  (4, 13, 23, 35)
 (∗) 19*. S. atrofuscum* (Schimp.) Limpr.  (16)
 (∗)* 20. S. brunnescens* Limpr.  (17)
 (∗)* 21. S. confertum* (Funck) Bruch & Schimp.  (3, 5, 20)
 (∗)* 22. S. crassipilum* H. H. Blom  (17)
 (∗) (∗∗)* 23. S. dupretii* (Thér.) W. A. Weber  (12)
 (∗)* 24. S. elegantulum* H. H. Blom  (10, 12)
 (∗)* 25. S. pruinosum* (Wilson ex Schimp.) G. Roth  (2)
 
**Dicranales H. Philib. ex M. Fleisch **
 
**Fissidentaceae Schimp. **
 
**7.* Fissidens* Hedw.**
 
*26. Fissidens taxifolius* Hedw.  (12)
 
**Ditrichaceae Limpr.**
 
**8.* Ceratodon* Brid.**
 
*27. Ceratodon purpureus* (Hedw.) Brid.  (6)
 
**9.* Distichium* Bruch & Schimp.**
 
*28. Distichium capillaceum* (Hedw.) Bruch & Schimp.  (2)
 
**10.* Ditrichum* Timm ex Hampe**
 
*29. Ditrichum flexicaule* (Schwägr.) Hampe  (16, 33)
 
**Dicranaceae Schimp.**
 
**11.* Dicranella* (Müll. Hal.)**
 
*30. Dicranella howei* Renauld & Cardot  (19)
 
*31. D. varia* (Hedw.) Schimp.  (2, 20, 23)
 
**12.* Dicranum *(Müll. Hal.) **
 
*32. Dicranum majus* Sm.  (6)
 (∗)* 33. D. polysetum* Sw. ex anon.  (12)
 
*34. D. tauricum* Sapjegin  (9, 23, 27)
 
**Pottiales M. Fleisch **
 
**Pottiaceae Schimp. **
 
**13.* Eucladium* Bruch & Schimp. **
 
*35. Eucladium verticillatum* (With.) Bruch & Schimp.  (1, 2)
 
**14.* Gymnostomum* Nees & Hornsch.**
 (∗)* 36. Gymnostomum aeruginosum* Sm.  (2)
 (∗)** 15.* Gyroweisia* Schimp.**
 (∗)* 37. Gyroweisia tenuis* (Hedw.) Schimp.  (2)
 
**16.* Tortella* (Müll. Hal.) Limpr.**
 (∗) (∗∗)* 38.  Tortella bambergeri* (Schimp.) Broth.  (22)
 (∗)* 39. T. humilis* (Hedw.) Jenn.  (9, 12)
 
*40. T. inclinata* (R. Hedw.) Limpr.  (16)
 
*41. T. squarrosa* (Brid.) Lindb.  (2)
 
*42. T. tortuosa* (Hedw.) Limpr (2, 6, 9, 12, 16, 20, 23, 37)
 
**17.* Trichostomum* Bruch **
 
*43. Trichostomum brachydontium* Bruch  (20, 31)
 
**18.* Weissia* Hedw. **
 (∗) (∗∗)* 44. Weissia condensa* var.* armata* (Thér. & Trab.) M. J. Cano, Ros & J. Guerra  (10)
 
*45. W. controversa* var.* controversa* Hedw.  (33)
 
**19.* Barbula* Hedw. **
 
*46. Barbula convoluta* var.* convoluta* Hedw.  (1, 12, 19)
 (∗)* 47. B. convoluta* var.* sardoa* Schimp.  (1, 12)
 (∗) (∗∗∗)* 48. B. enderesii* Garov.  (21)
 
*49. B. unguiculata* Hedw.  (1, 2, 5, 28)
 
**20.* Bryoerythrophyllum* P. C. Chen **
 
*50. Bryoerythrophyllum recurvirostrum* (Hedw.) P. C. Chen  (2, 26)
 (∗)* 51. B. rubrum* (Jur. ex Geh.) P. C. Chen  (21)
 (∗)** 21.* Cinclidotus* P. Beauv.**
 (∗)* 52. Cinclidotus danubicus* Schiffn. & Baumgartner  (2)
 (∗)* 53. C. riparius* (Host ex Brid.) Arn.  (2)
 
**22.* Crossidium* Jur. **
 
*54. Crossidium squamiferum* (Viv.) Jur.  (28)
 
**23.* Didymodon* Hedw. **
 
*55. Didymodon acutus* (Brid.) K. Saito  (2, 7, 8)
 
*56. D. fallax* (Hedw.) R. H. Zander  (1)
 (∗)* 57. D. ferrugineus* (Schimp. ex Besch.) M. O. Hill  (3, 12)
 (∗)* 58. D. luridus* Hornsch.  (12, 33)
 
*59. D. nicholsonii* Culm.  (12, 33)
 (∗)* 60. D. rigidulus* Hedw.  (8)
 (∗)* 61. D. tomaculosus* (Blockeel) M. F. V. Corley (20) published as Asia record (Canlı & Çetin, 2012)
 (∗)* 62. D. tophaceus *(Brid.) Lisa  (1, 35)
 
*63. D. vinealis* (Brid.) R. H. Zander  (1, 21, 34)
 
**24.* Pseudocrossidium* R. S. Williams **
 (∗)* 64. Pseudocrossidium hornschuchianum* (Schultz) R. H. Zander  (5, 34)
 (∗)* 65. P. revolutum* (Brid.) R. H. Zander  (34)
 
**25.* Syntrichia* Brid. **
 (∗)* 66. Syntrichia calcicola* J. J. Amann  (13, 22, 25)
 
*67. S. caninervis* Mitt.  (8)
 (∗)* 68. S. laevipila* Brid.  (8)
 (∗) 69*. S. montana* Nees  (1, 2, 8, 13)
 
*70. S. norvegica* F. Weber  (17)
 (∗)* 71. S. ruralis* var.* ruraliformis* (Besch.) Delogne  (14, 26, 27, 33, 35)
 
*72. S. ruralis* var.* ruralis* (Hedw.) F. Weber & D. Mohr  (3, 17, 23, 27, 34, 36, 37)
 
*73. S. virescens* (De Not.) Ochyra  (2)
 
**26.* Tortula* Hedw. **
 (∗)* 74. Tortula brevissima* Schiffn.  (2)
 (∗)* 75. T. canescens* Mont.  (37)
 
*76. T. inermis* (Brid.) Mont.  (2, 10)
 (∗)* 77. T. lindbergii* Broth.  (2)
 
*78. T. muralis* Hedw.  (2, 3, 15, 17, 21, 32)
 (∗) 79*. T. schimperi* M. J. Cano, O. Werner & J. Guerra  (21, 23, 29, 37)
 
*80. T. subulata* Hedw.  (9, 12, 21, 37)
 (∗)* 81. T. vahliana* (Schultz) Mont.  (11, 28)
 
**Orthotrichales Dixon **
 
**Orthotrichaceae Arn. **
 
**27.* Orthotrichum* Hedw. **
 
*82. Orthotrichum anomalum* Hedw.  (2, 8, 26, 33)
 (∗)* 83. O. cupulatum* Hoffm. ex Brid.  (2, 9, 12)
 
*84. O. urnigerum* Myrin  (2, 33)
 
*85. O. diaphanum* Schrad. ex Brid.  (2, 33)
 
*86. O. pallens* Bruch ex Brid.  (2, 8, 11, 12)
 (∗)* 87. O. rupestre* Schleich. ex Schwägr.  (36)
 
*88. O. affine* Schrad. ex Brid  (2)
 (∗)* 89. O. speciosum* Nees  (3, 11, 12)
 
*90. O. striatum* Hedw.  (3)
 
**Hedwigiales Ochyra **
 (∗)** Hedwigiaceae Schimp. **
 (∗)** 28.* Hedwigia* P. Beauv.**
 (∗) (∗∗)* 91. Hedwigia ciliata* var.* leucophaea* Bruch & Schimp.  (3)
 
**Bryales Limpr. **
 
**Bartramiaceae Schwägr. **
 
**29.* Philonotis* Brid. **
 (∗)* 92. Philonotis caespitosa* Jur.  (22)
 
*93. P. fontana* (Hedw.) Brid.  (15)
 (∗)* 94. P. seriata* Mitt.  (17)
 
**Bryaceae Schwägr. **
 
**30.* Bryum* Hedw. **
 
*95. Bryum argenteum* Hedw.  (12, 17, 37)
 (∗)* 96. B. funkii* Schwägr.  (13)
 (∗)* 97. B. schleicheri* DC.  (22)
 (∗)* 98. B. subapiculatum* Hampe  (20)
 (∗)* 99. B. weigelii* Spreng.  (22)
 
**31.* Imbribryum* N. Pedersen **
 
*100. Imbribryum alpinum* Huds. ex With.  (11)
 (∗)* 101. I. mildeanum* (Jur.) J. R. Spence  (1)
 
**32.* Ptychostomum* Hornsch. **
 (∗)* 102. Ptychostomum borreale* (F. Weber & D. Mohr) Ochyra & Bednarek-Ochyra (2)
 
*103. P. capillare* (Hedw.) Holyoak & N. Pedersen  (12, 20)
 (∗)* 104. P. cernuum* (Hedw.) Hornsch.  (2)
 (∗) 105.* P. creberrimum* (Taylor) J. R. Spence & H. P. Ramsay  (21)
 
*106. Ptychostomum imbricatulum* (Müll. Hal.) Holyoak & N. Pedersen  (20)
 (∗)* 107. P. moravicum* (Podp.) Ros & Mazimpaka (8, 9, 20)
 
*108. P. pallens* (Sw.) J. R. Spence  (1, 12, 15)
 
*109. P. pseudotriquetrum* (Hedw.) J. R. Spence & H. P. Ramsay  (3)
 
**Mielichhoferiaceae Schimp. **
 
**33.* Pohlia* Hedw. **
 
*110. Pohlia cruda* (Hedw.) Lindb.  (20)
 (∗)* 111. P. ludwigii* (Spreng. ex Schwägr.) Broth.  (27)
 
**Mniaceae Schwägr. **
 
**34.* Mnium* Hedw. **
 (∗)* 112. Mnium stellare* Hedw.  (20)
 
**Cinclidiaceae Kindb. **
 
**35.* Rhizomnium* (Broth.) T. J. Kop. **
 
*113. Rhizomnium punctatum* (Hedw.) T. J. Kop.  (13)
 
**Plagiomniaceae T. J. Kop. **
 
**36.* Plagiomnium* T. J. Kop. **
 (∗)* 114. Plagiomnium affine* (Blandow ex Funck) T. J. Kop.  (12)
 
*115. P. elatum* (Bruch & Schimp.) T. J. Kop.  (2)
 (∗)* 116. P. medium* (Bruch & Schimp.) T. J. Kop.  (2)
 
**Hypnales (M. Fleisch.) W. R. Buck & Vitt **
 (∗)** Fontinalaceae Schimp.**
 (∗)** 37.* Fontinalis* Hedw.**
 (∗)* 117. Fontinalis antipyretica* subsp.* antipyretica* Hedw.  (2)
 (∗)* 118. F. antipyretica* subsp.* gracilis* (Lindb.) Kindb.  (2)
 (∗)* 119. F. hypnoides* var.* hypnoides* C. Hartm.  (2)
 
**Amblystegiaceae Kindb. **
 
**38.* Amblystegium* Schimp. **
 
*120. Amblystegium serpens* (Hedw.) Schimp.  (1,2)
 (∗)** 39.* Campyliadelphus* (Kindb.) R. S. Chopra**
 (∗)* 121. Campyliadelphus chrysophyllus* (Brid.) R. S. Chopra  (12)
 (∗)* 122. C. elodes* (Lindb.) Kanda  (1)
 
**40.* Cratoneuron* (Sull.) Spruce **
 
*123. Cratoneuron filicinum* (Hedw.) Spruce  (2, 13, 17, 21, 22)
 
**41.* Hygroamblystegium* Loeske **
 
*124. Hygroamblystegium fluviatile* (Hedw.) Loeske  (1)
 (∗)* 125. H. tenax* (Hedw.) Jenn.  (2)
 
*126. H. varium* (Hedw.) Mönk.  (15)
 (∗)* 127. H. varium* var.* humile* (P. Beauv.) Vanderp. & Hedenäs  (1, 2, 12)
 (∗)** 42.* Hygrohypnum* Lindb. **
 (∗)* 128. Hygrohypnum luridum* (Hedw.) Jenn.  (22)
 
**43.* Palustriella* Ochyra **
 
*129. Palustriella commutata* (Hedw.) Ochyra  (2, 15, 37)
 (∗)* 130. P. decipiens* (De Not.) Ochyra  (2)
 
*131. P. falcata* (Brid.) Hedenäs  (2)
 
**44.* Pseudocampylium* Vanderp. & Hedenäs**
 (∗)* 132. Pseudocampylium radicale *(P. Beauv.) Schimp.  (2, 4)
 
**Leskeaceae Schimp. **
 
**45* Pseudoleskeella* Kindb. **
 
*133. Pseudoleskeella catenulata* (Brid. ex Schrad.) Kindb  (4)
 (∗)* 134. P. nervosa* (Brid.) Nyholm  (4)
 (∗)* 135. P. tectorum* (Funck ex Brid.) Kindb. ex Broth.  (9)
 
**Thuidiaceae Schimp. **
 
**46.* Abietinella* Kindb. **
 
*136. Abietinella abietina* var.* abietina* (Hedw.) M. Fleisch.  (2)
 
*137. A. abietina* var. hystricosa (Mitt.) Sakura  (3, 12)
 
**47.* Thuidium* Schimp. **
 
*138. Thuidium delicatulum* (Hedw.) Schimp.  (35)
 
**Brachytheciaceae Schimp. **
 
**48.* Pseudoscleropodium* Schimp. **
 
*139. Pseudoscleropodium purum* (Hedw.) M. Fleisch.  (12)
 
**49.* Plasteurhynchium* M. Fleisch. ex Broth. **
 (∗)* 140. Plasteurhynchium striatulum* (Spruce) M. Fleisch.  (13)
 
**50.* Rhynchostegium* Schimp. **
 
*141. Rhynchostegium alopecuroides* (Brid.) A. J. E. Sm.  (13, 15)
 
*142. R. confertum* (Dicks.) Schimp.  (31)
 (∗)* 143. R. megapolitanum* (Blandow ex F. Weber & D. Mohr) Schimp.  (1)
 
*144. R. riparioides* (Hedw.) Cardot  (1, 2)
 
**51.* Oxyrrhynchium* (Schimp.) Warnst **
 
*145. Oxyrrhynchium hians* (Hedw.) Loeske  (13)
 (∗)* 146. O. speciosum* (Brid.) Warnst.  (2, 12)
 
**52.* Kindbergia* Ochyra **
 
*147. Kindbergia praelonga* (Hedw.) Ochyra  (9, 11, 12)
 
**53.* Sciuro-hypnum* Hampe **
 (∗)* 148. Sciuro-hypnum latifolium* Ignatov & Huttunen  (2, 15, 17)
 (∗)* 149. S. reflexum* (Starke) Ignatov & Huttunen  (11)
 
**54.* Brachythecium* Schimp. **
 
*150. Brachythecium albicans* (Hedw.) Schimp.  (11, 19)
 (∗)* 151. B. geheebii* Milde  (4, 11)
 
*152. B. glareosum* (Bruch ex Spruce) Schimp.  (19)
 
*153. B. rivulare* Schimp.  (2)
 
**55.* Eurhynchiastrum* Ignatov & Huttunen **
 
*154. Eurhynchiastrum pulchellum* (Hedw.) Ignatov & Huttunen  (20, 23)
 
**56.* Brachytheciastrum* Ignatov & Huttunen **
 
*155. Brachytheciastrum velutinum* (Hedw.) Ignatov & Huttunen  (20, 21)
 
**57.* Homalothecium* Schimp. **
 (∗)* 156. Homalothecium aureum* (Spruce) H. Rob.  (5)
 
*157. H. lutescens* (Hedw.) H. Rob.  (3, 12, 14, 28, 35)
 
*158. H. philippeanum* (Spruce) Schimp.  (21, 30)
 
*159. H. sericeum* (Hedw.) Schimp.  (1, 2, 13, 20, 23)
 
**Hypnaceae Schimp. **
 
**58.* Calliergonella* Loeske **
 
*160. Calliergonella cuspidata* (Hedw.) Loeske  (15, 17)
 (∗)** 59.* Campylophyllum* (Schimp.) M. Fleisch. **
 (∗)* 161. Campylophyllum sommerfeltii* (Myrin) Hedenäs  (2)
 
**60.* Homomallium* (Schimp.) Loeske **
 
*162. Homomallium incurvatum* (Schrad. ex Brid.) Loeske  (4)
 
**61.* Hypnum* Hedw. **
 
*163. Hypnum andoi* A. J. E. Sm.  (3, 6, 7, 11, 12, 20)
 (∗)* 164. H. bambergeri* Schimp.  (14)
 
*165. H. cupressiforme var. cupressiforme* Hedw.  (2, 20, 24)
 
*166. H. cupressiforme var. lacunosum* Brid.  (9, 34)
 (∗)* 167. H. cupressiforme var. subjulaceum* Molendo  (14)
 
*168. H. imponens* Hedw.  (12)
 (∗)* 169. H. recurvatum* (Lindb. & Arnell) Kindb.  (26)
 (∗)* 170. H. revolutum* (Mitt.) Lindb.  (20)
 
**62.* Pylaisia* Schimp. **
 
*171. Pylaisia polyantha* (Hedw.) Schimp.  (9)
 (∗)** 63.* Taxiphyllum* M. Fleisch.**
 (∗)* 172. Taxiphyllum wissgrillii* (Garov.) Wijk & Margad.  (20)
 
**Pterigynandraceae Schimp. **
 
**64.* Heterocladium* Schimp. **
 (∗)* 173. Heterocladium dimorphum* (Brid.) Schimp.  (9)
 
**65.* Pterigynandrum* Hedw. **
 
*174. Pterigynandrum filiforme* Hedw.  (20, 21, 22)
 (∗)** Pylaisiadelphaceae Goffinet & W. R. Buck**
 (∗)** 66.* Platygyrium* Schimp.**
 (∗)*  175.  Platygyrium repens* (Brid.) Schimp.  (20)
 (∗)** Cryphaeaceae Schimp.**
 (∗)** 67.* Cryphaea* D. Mohr**
 (∗)*  176. Cryphaea heteromalla* (Hedw.) D. Mohr  (4)
 
**Leucodontaceae Schimp. **
 
**68.* Leucodon* Schwägr. **
 
*177. Leucodon sciuroides* (Hedw.) Schwägr.  (4, 8, 20, 23, 30)
 
**Neckeraceae Schimp. **
 
**69.* Neckera* Hedw. **
 (∗)* 178. Neckera menziesii* Drumm.  (23, 30)

A total of 178 taxa belonging to 69 genera and 26 families were determined following the identification of 1500 specimens collected from 37 localities between 2009 and 2011. Of these, 94 taxa are new for the A3 square. The location data of* Grimmia crinitoleucophaea* Cardot and* Barbula enderesii* Garov. are the first records for Turkey, and* Encalypta spathulata* Müll. Hal.,* Schistidium dupretii* (Thér.) W. A. Weber,* Weissia condensa* var.* armata* (Thér. & Trab.) M. J. Cano, Ros & J. Guerra,* Tortella bambergeri* (Schimp.),* Barbula enderesii* Garov.,* Hedwigia ciliata* var.* leucophaea* Bruch & Schimp., and* Campyliadelphus elodes* (Lindb.) Kanda are recorded for the second time.

*Grimmia crinitoleucophaea Cardot*. The basal marginal cells of the perichaetial and subperichaetial leaves are hyaline. Setae are very short and sporophytes are hidden between perichaetial leaves. 

*Encalypta spathulata Müll. Hal.* Plants form an extensive mat and are covered with a mass of pale-colored calyptrae. The rostrum of the calyptra is short. Seta is red to dark red and quite fragile. Leaves are narrow and irregularly twisted, with a shiny dark-brown costa. 

*Barbula enderesii Garov.* Plants have strongly differentiated convolute perichaetial leaves. Setae are mostly yellow and annulus is strongly differentiated. Leaves are strongly falcate with dense high papillae. 

*Schistidium dupretii (Thér.) W. A. Weber.* Plants are small and form low tufts. The central strand is distinct and hair-point is very short. Costa and leaf margins are smooth. Sporophytes are common. Peristome teeth are red and entire. 

*Weissia condensa var. armata (Thér. & Trab.) M. J. Cano, Ros & J. Guerra.* Plants are almost 0.5 cm. Leaves are patent and margins are slightly incurved. Upper laminal cells are slightly papillose. 

*Hedwigia ciliata var. leucophaea Bruch & Schimp.* Differs from* H. ciliata* var.* ciliata* in the length of the hair-point (7–33% versus 22–65% of leaf length), in having less strongly papillose hair-points, and in having more strongly recurved stem leaf margins. 

*Tortella bambergeri (Schimp.).* The long, narrow leaves are slightly curved or almost straight when moist and dry to a contorted spiral. They have plane margins. The upper part of the leaf is very fragile and most stems in a tuft have only a few leaf tips present. Transverse section of stem has a distinct central strand. 

*Campyliadelphus elodes (Lindb.) Kanda.* The plants are rather slender, with irregularly and rather loosely branched, dull green or yellow-green shoots. Shoots reach 4-5 cm or more in length. Leaves are acute and gradually tapering from base to apex. Leaf margins are obscurely denticulate above. Costa is long and single and is usually extending the apex or near the apex.

The dominant family in the study area is Pottiaceae (48 taxa). Other families with the highest number of taxa are, respectively, Brachytheciaceae (21), Grimmiaceae (17), and Bryaceae (15). The most species-rich genera recorded were* Grimmia* (9),* Didymodon* (9),* Tortula* (9), and* Orthotrichum* (9). Acrocarpous mosses constitute 65% and pleurocarpous-mosses constitute 35%.

The data from this survey was compared to studies from neighboring areas (Table 2) [40–43] and shows that the number of taxa of Pottiaceae family members, which display acrocarpous mosses in the Mediterranean, is higher than from other regions. These findings are similar to seed plant vegetation surveys of the area. This is due to the wet and mild climate, large number of microhabitats and enclaves, and different ecological conditions present in the region.

It is hoped that further studies will contribute more species to the knowledge of moss flora of Turkey and that this study will be useful as a guide for future research.

## Figures and Tables

**Table 1:** Vegetations and coordinate information of stations.

Station	Altitude (m)	Coordinate	Dominant vegetation
1	498	N 40°41.154′/E 035°57,471′	*Pinus nigra* subsp. *pallasiana *
2	872	N 40°41.006′/E 036°3,902′	*Quercus robur* and *Populus tremula *
3	845	N 40°53.685′/E 035°46,626′	*Quercus robur* and *Pinus nigra *subsp. *pallasiana *
4	1020	N 40°54.252′/E 035°52,544′	*Fagus orientalis *
5	870	N 40°54.329′/E 035°58,941′	Reeds and agricultural land
6	1070	N 40°51.936′/E 036°10,093′	*Pinus sylvestris* and *Fagus orientalis *
7	492	N 40°45.577′/E 036°6,736′	*Pinus brutia *
8	565	N 40°44.784′/E 035°59,210′	*Pinus brutia* and *Quercus hartwissiana *
9	1330	N 40°46.296′/E 035°59,988′	*Juniperus nana* and *Pinusnigra* subsp. *pallasiana *
10	903	N 40°46.289′/E 036°6,915′	*Juniperus nana* and *Pinusnigra* subsp. *pallasiana *
11	1080	N 40°50.550′/E 035°47,292′	*Quercus hartwissiana *
12	1050	N 40°51.909′/E 035°47,648′	*Pinus nigra* subsp. *pallasiana *
13	1100	N 40°50.738′/E 035°50,303′	*Pinus nigra* subsp. *pallasiana *
14	1430	N 40°50.507′/E 035°54,296′	*Pinus nigra* subsp. *pallasiana *
15	1240	N 40°48.839′/E 035°57,754′	*Pinus nigra* subsp. *pallasiana* and *Astragalus *sp.
16	1180	N 40°47.305′/E 036°2,430′	*Pinus nigra* subsp. *pallasiana* and * Juniperus oxycedrus *
17	1750	N 40°46.322′/E 035°54,672′	High mountain meadows
18	2040	N 40°46.786′/E 035°55,621′	*Astragalus* sp. and *Acantholimon* sp.
19	227	N 40°45.304′/E 036°19,371′	*Quercus hartwissiana *
20	1060	N 40°48.249′/E 036°9,532′	*Cedrus libani*, *Pinus nigra* subsp. *pallasiana,* and *Fagus orientalis *
21	1230	N 40°48.080′/E 036°7,876′	*Cedrus libani* and *Pinus nigra* subsp. *pallasiana *
22	1550	N 40°47.935′/E 036°6,600′	*Pinus nigra* subsp. *pallasiana, Astragalus *sp., and *Euphorbia *sp.
23	1650	N 40°48.358′/E 036°5,140′	*Pinus nigra* subsp. *Pallasiana *
24	1320	N 40°47.520′/E 036°7,948′	*Pinus nigra* subsp. *pallasiana, Euphorbia *sp., and *Juniperus nana *
25	664	N 40°46.043′/E 036°7,207′	*Pinus brutia* and *Quercus hartwissiana *
26	1320	N 40°50.614′/E 035°53,952′	*Salix alba *
27	1570	N 40°49.876′/E 035°55,810′	*Astragalus* sp. and *Acantholimon* sp.
28	712	N 40°51.319′/E 036°9,434′	*Pinus sylvestris* and *Fagus orientalis *
29	943	N 40°52.037′/E 036°5,651′	*Carpinus betulus *
30	1020	N 40°52.331′/E 035°48,586′	*Pinus sylvestris* and *Fagus orientalis *
31	483	N 40°45.227′/E 035°36,509′	Agricultural land
32	905	N 40°41.896′/E 036°3,180′	*Pinus brutia* and *Quercus hartwissiana *
33	1110	N 40°38.948′/E 035°57,307′	*Pinus nigra* subsp.* pallasiana *
34	513	N 40°41.498′/E 035°51,422′	*Pinus nigra* subsp.* pallasiana *
35	1520	N 40°52.581′/E 035°54,388′	*Pinus nigra* subsp.* pallasiana *
36	1210	N 40°45.033′/E 035°52,059′	*Quercus hartwissiana *
37	1890	N 40°49.850′/E 036°3,666′	*Astragalus* sp. and *Acantholimon* sp.

**Table 2:** The comparison of the taxa distribution according to the families.

	Amasya Akdağ	Cankırı Gürgenli Mountain	Ilgaz Yenice Forests	Ilgaz Mountain National Park	Çankırı Eldivan Mountain
Pottiaceae	27	16,9	15,6	13,7	23,3
Brachytheciaceae	11,6	20,5	12,1	13,7	18,3
Grimmiaceae	9,4	7,2	10	6,4	8,3
